# Orthodontic Management in Pediatric Patients with Rare Diseases: Case Reports

**DOI:** 10.3390/jcm14010055

**Published:** 2024-12-26

**Authors:** Valeria Luzzi, Miriam Fioravanti, Lilia Mitrano, Beatrice Marasca, Matteo Saccucci, Mauro Celli, Luca Celli, Iole Vozza, Gaetano Ierardo

**Affiliations:** 1 Department of Oral and Maxillo-Facial Sciences, Sapienza University of Rome, U.O.C. Pediatric Dentistry Unit, 00161 Rome, Italybeatrice.marasca@uniroma1.it (B.M.); iole.vozza@uniroma1.it (I.V.);; 2 Department of Pediatrics, Sapienza University of Rome, 00161 Rome, Italy

**Keywords:** orthodontic miniscrew, osteogenesis imperfecta, ectodermal dysplasia, maxillary skeletal expander (MSE), pediatric dentistry, case reports, orthodontic management

## Abstract

**Background**: The orthodontic management of pediatric patients with rare diseases, such as Ectodermal Dysplasia (ED) and Osteogenesis Imperfecta (OI), requires complex protocols due to dental anomalies in both the number and structure of teeth. These conditions necessitate a departure from traditional orthodontic approaches, as skeletal anchoring is often required because of these anomalies. **Case Presentation:** A patient with ED, characterized by hypodontia and malformed teeth, presented with insufficient natural teeth for anchorage. This challenge was addressed using a Maxillary Skeletal Expander (MSE) with miniscrews. Cone-beam computed tomography (CBCT) and cephalometric radiographs were used to assess bone density, which guided the creation of a customized hybrid device. A second patient with OI, a condition causing fragile bones, had malformed teeth and a high risk of fractures. Skeletal anchoring with MSE and miniscrews was chosen to avoid damaging brittle bones. The fragile nature of the patient’s bones required careful planning and close monitoring throughout the treatment process. Both patients were treated at the UOC of Pediatric Dentistry, Sapienza University of Rome, using MSE with miniscrews. Pre- and post-treatment imaging (CBCT and cephalometric radiographs) were used to evaluate bone quality and monitor progress. Skeletal anchoring successfully addressed the unique challenges in both cases, achieving outcomes comparable to those in unaffected patients. **Discsussions**: despite limited bone volume, MSE successfully achieved maxillary arch expansion and improved occlusion. Post-treatment radiographs showed successful maxillary expansion and alignment without complications. **Conclusions:** This case series highlighted the effectiveness of MSE with miniscrews in treating patients with rare diseases. It advances orthodontic management by offering reliable solutions for complex cases involving dental anomalies and compromised bone structures.

## 1. Introduction

Ectodermal Dysplasia (ED) is a group of hereditary conditions that affect ectodermal structures such as teeth, hair, and nails. These conditions lead to oral manifestations such as oligodontia, anodontia, enamel defects, and malformed teeth. These dental anomalies present significant orthodontic challenges due to the lack of sufficient teeth for anchorage and the need for complex treatment planning. ED patients often face compromised facial aesthetics, which can negatively impact self-esteem, making early intervention critical for improving both functional and psychological outcomes [1,2,3,4].

Osteogenesis Imperfecta (OI) is a genetic disorder characterized by bone fragility due to defects in collagen synthesis, leading to skeletal deformities and an increased risk of fractures. OI patients often exhibit malocclusions and abnormal dentin formation (Dentinogenesis Imperfecta), complicating orthodontic treatment. Additionally, bisphosphonate therapy, commonly prescribed to strengthen bones, inhibits orthodontic tooth movement, requiring careful consideration when planning treatment. The fragile bone structure in OI necessitates a multidisciplinary approach and careful monitoring to avoid complications during orthodontic therapy [5,6,7,8].

Both conditions present unique challenges in orthodontic management, particularly concerning anchorage, dental movement, and patient cooperation. Skeletal anchoring methods, such as the use of temporary anchorage devices (TADs), offer reliable solutions in these complex cases, enabling effective treatment even with limited natural tooth support [9,10,11,12,13].

A recent review of the literature analyzes the effect and stability of miniscrew-assisted rapid palatal expansion (MARPE) for the clinical treatment of patients with maxillary transverse deficits (MTD). The authors demonstrated that MARPE can be an effective treatment for patients with MTD, ensuring transverse skeletal expansion even in late adolescence. Furthermore, MARPE presents fewer periodontal side effects compared to conventional rapid palatal expanders (RPE) and offers numerous clinical advantages [14,15,16,17,18,19].

This case series aims to highlight innovative orthodontic approaches for addressing complex skeletal malocclusions in pediatric patients with rare conditions.

For these reasons, we chose to use miniscrew-assisted rapid palatal expansion (MARPE) with modified or hybrid TAD-assisted expanders that incorporate both dental and skeletal anchorage in pediatric patients with special needs. This study described the orthodontic management of pediatric patients with systemic diseases associated with widespread agenesis or structural defects in enamel and dentin, such as osteogenesis imperfecta and ectodermal dysplasia.

## 2. Case Presentation

At the Complex Operative Unit (UOC) of Pediatric Dentistry, Department of Oral and Maxillofacial Sciences, Sapienza University of Rome, Policlinico Umberto I, two pediatric patients of similar age, both in mixed dentition, were treated for significant maxillofacial alterations and anchorage deficiencies caused by systemic pathologies with oral manifestations, specifically Ectodermal Dysplasia (ED) and Osteogenesis Imperfecta (OI). Both conditions presented unique orthodontic challenges due to dental anomalies and bone fragility, requiring a specialized approach combining a Maxillary Skeletal Expander (MSE) with skeletal anchoring.

Cases were selected based on the following inclusion criteria: patients aged 6–12 years, with a confirmed diagnosis of ED or OI, exhibiting significant maxillofacial abnormalities (such as oligodontia, anodontia, or malformed teeth), and requiring maxillary expansion due to anchorage deficiencies. Exclusion criteria included patients with severe systemic health conditions unrelated to ED or OI, previous orthodontic treatment, or insufficient bone quality to support skeletal anchoring.

Informed written consent was obtained from each patient’s parents. A thorough pre-treatment assessment was conducted, which included systemic and physiological anamnesis, assessment of the patients’ degree of cooperation, and collaboration with the pediatrician to review any ongoing pharmacological therapies related to the systemic pathologies. The pediatrician’s collaboration was crucial for evaluating potential contraindications to orthodontic treatment, particularly due to the fragility of the patients’ bones in OI.

The treatment involved two clinical phases: the surgical phase (miniscrew insertion) and the orthodontic phase (activation of the MSE).

**Figure 1:**
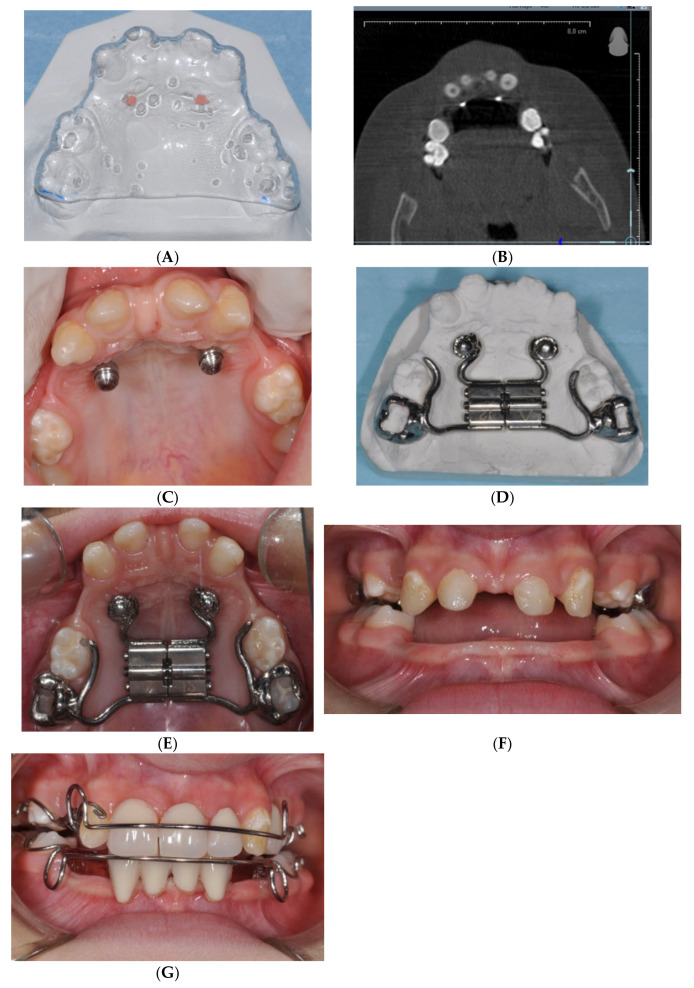
(**A**) Customized surgical guides for hypihydrotic ED patient. (**B**) CBCT scan performed for evaluation of bone quality and quantity for hypihydrotic ED patient. (**C**) Miniscrew placement in hypihydrotic ED patient. (**D**) Customized hybrid MSE for hypihydrotic ED patient. (**E**) MSE application for hypihydrotic ED patient. (**F**) MSE application for hypihydrotic ED patient. (**G**) Design and application of Schwarz appliances incorporated with dental elements in ED patient.

The initial diagnostic workup included clinical examination, radiographs (orthopantomograms and lateral cephalometric radiographs), and cephalometric analysis for skeletal assessment. Alginate and silicone impressione were taken for study models and for fabricating a customized hybrid MSE. A CBCT scan was performed to evaluate bone quality and quantity and to design customized surgical guides (Figure 1A) for the precise placement of the miniscrews (Figure 1B).

The surgical procedure involved local anesthesia with a vasoconstrictor and the use of a thermoplastic template with two palatal holes filled with gutta-percha to guide miniscrew placement. Using this template, perforations were made with a rosette bur, followed by the use of a helical drill (0.9 mm or 1.1 mm) to perforate the first 2–3 mm of cortical bone. Miniscrews (Imtec ORTHO Implant^®^, 6 mm length, 1.8 mm diameter) were then manually inserted using a self-tapping screwdriver. The screw insertion was performed with careful attention to avoid excessive torsional loads, ensuring primary stability for immediate loading. Following insertion, the screws were immediately loaded with forces ranging from 50 g to 250 g (Figure 1C).

This study was approved by the Institutional Review Board (IRB) of the Pediatric Dentistry Department at Sapienza University of Rome, ensuring adherence to the ethical standards outlined in the Declaration of Helsinki for clinical research. Informed consent was obtained from the legal guardians of all patients, who were thoroughly informed about the risks and benefits of the proposed treatment.

The MSE was cemented with glass ionomer cement (Figure 1E,F), and the expansion protocol involved two activations per day for the first 15 days [13]. The parents were provided with detailed instructions on how to perform these activations. The expander was retained in place for six months after the active phase to allow for stabilization. The progress of the treatment was monitored through regular follow-ups, including occlusal radiographs to assess the opening of the midpalatal suture at the end of active therapy. Follow-up radiographs were also taken one year after completion of the expansion to evaluate midpalatal suture reossification and reorganization, ensuring the long-term success of the therapy.

Treatment success was defined by the following criteria: successful expansion of the maxillary arch, proper alignment of the teeth, absence of complications (such as bone fractures or screw displacement), and stabilization of the midpalatal suture post-treatment. Radiographic and clinical evaluations were performed at key stages to ensure the objectives were met.

Several challenges were encountered during the treatment process. In patients with OI, the fragility of the bone increased the risk of screw instability or displacement. To mitigate this, smaller diameter screws were used, and a detailed assessment of bone density was performed using CBCT before screw insertion. In the case of ED, the lack of sufficient natural teeth for conventional anchorage posed another difficulty. This was addressed by using the hybrid MSE system, combining dental anchorage on the upper first molars and skeletal anchorage via miniscrews. Moreover, the lack of patient cooperation, due to age-related factors, was mitigated by educating both the patients and their parents on the importance of adherence to the treatment protocol, which improved overall compliance.

### 2.1. MSE Treatment in Patient with Ectodermal Dysplasia

Ectodermal Dysplasia (ED) is a group of hereditary conditions with a prevalence of approximately 1 in 15,000 individuals, characterized by abnormalities in two or more ectodermal structures, such as hair, teeth, nails, sweat glands, craniofacial structures, and fingers. Patients with ED typically present with sparse and brittle hair, a prominent forehead, pronounced chin, enlarged nose, and abnormal eye development. The oral manifestations are often severe, including multiple agenesis of both deciduous and permanent teeth, enamel defects, and malformed teeth.

**Figure 2:**
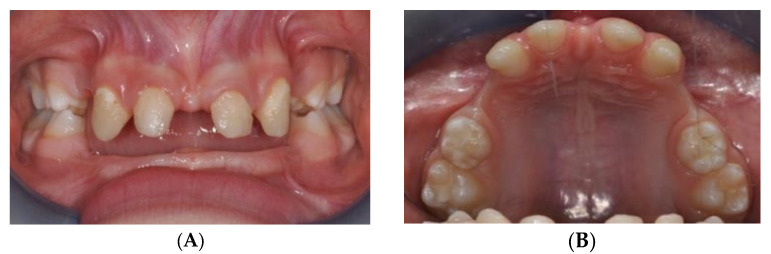
(**A**) Frontal Intraoral examination of hypihydrotic ED patient. (**B**) Intraoral examination of hypihydrotic ED patient.

An 8-year-old male patient with hypohydrotic ED and oral respiration presented to the Complex Operative Unit (UOC) of Pediatric Dentistry, Department of Oral and Maxillofacial Sciences, Sapienza University of Rome. The patient exhibited typical extraoral features, including a brachyfacial type, small chin, acute nasolabial angle, protruded lips, prominent forehead, and large, low-set ears. Intraoral examination revealed multiple tooth agenesis and upper jaw hypotrophy, which was confirmed by orthopantomography (Figure 2A,B). Cephalometric analysis showed a mild tendency toward skeletal Class III, hypodivergence, and reduced anterior facial height.

The treatment plan was carefully designed to address the patient’s maxillary hypoplasia, dental agenesis, and specific craniofacial features. A hybrid Maxillary Skeletal Expander (MSE) was selected for orthodontic treatment, as it would provide the necessary skeletal anchorage for maxillary expansion. The choice of MSE was based on its ability to expand the maxillary arch and provide support for the placement of dental elements in subsequent stages of treatment.

Following the initial activation, the MSE was kept inactive for 9 months to allow bone apposition at the midpalatal suture site. After this period, two Schwarz appliances were fabricated and incorporated with dental elements to enhance both function and aesthetics (Figure 1G). The use of the Schwarz appliance was selected to assist in the eruption of permanent teeth while maintaining both aesthetic and functional integrity.

The patient reported mild discomfort during the early phases of treatment, especially during the activation of the MSE. However, the discomfort was manageable with pain relief and was temporary. The patient’s parents were actively involved in monitoring the expansion process, and regular follow-ups helped to address any concerns. The patient showed good compliance with treatment instructions, and both the patient and parents expressed satisfaction with the gradual improvement in occlusion and appearance (Figure 2A,B).

### 2.2. MSE Treatment in Patients with Osteogenesis Imperfecta and Dentinogenesis Imperfecta

Osteogenesis Imperfecta (OI) is a rare genetic disorder characterized by defects in type I collagen synthesis due to mutations in the COL1A1 and COL1A2 genes, inherited in an autosomal dominant manner. OI presents with increased skeletal fragility, decreased bone mass, and features such as ligamentous laxity, scoliosis, blue sclerae (a hallmark sign), hearing loss in adulthood, short stature, and associated Dentinogenesis Imperfecta (DI). DI is a hereditary disorder that results in abnormal dentin formation, affecting both deciduous and permanent dentition. Its prevalence is approximately 1 in 6000.

Bisphosphonates (BPs), which inhibit osteoclast activity and reduce bone turnover, are the standard treatment for OI and are often administered intravenously every three months from birth. While BPs significantly reduce fracture risk and improve bone density, they also pose dental challenges. By inhibiting osteoclast function, BPs can hinder orthodontic tooth movement, creating difficulties in orthodontic treatment for OI patients.

To address these challenges, a validated protocol has been developed at the Complex Operative Unit (UOC) of Pediatric Dentistry, Sapienza University of Rome, in collaboration with the Department of Pediatrics, Policlinico Umberto I. This protocol involves a temporary suspension of BP therapy, under the careful supervision of the pediatrician, for three months prior to initiating orthodontic treatments such as rapid maxillary expansion (RME). This strategy allows for effective palatal suture opening, physiological healing, and callus formation without interference from BP-induced inhibition of osteoclast activity.

**Figure 3:**
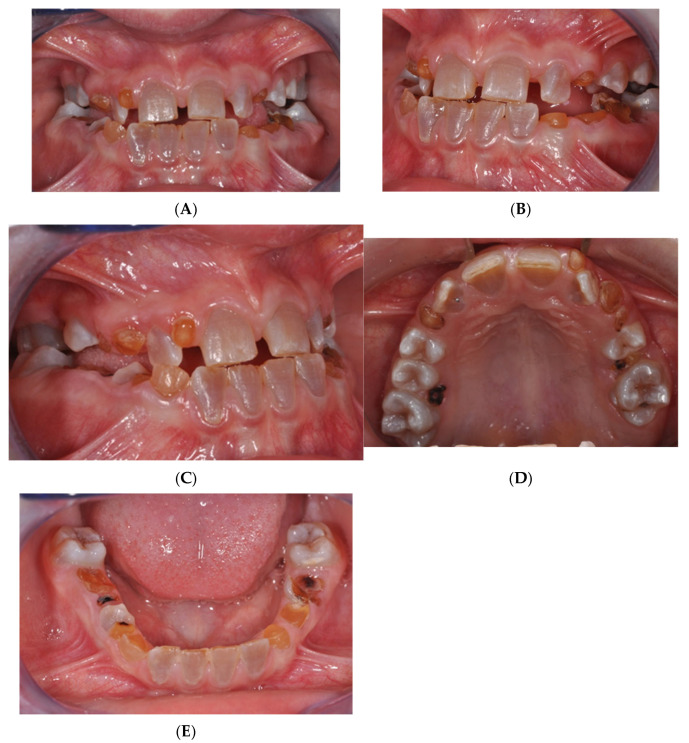
(**A**) Intraoral examination of OI patient. (**B**) Intraoral examination of OI patient. (**C**) Intraoral examination of OI patient. (**D**) Intraoral examination of OI patient. (**E**) Intraoral examination of OI patient.

A 9-year-old female patient diagnosed with OI and DI, receiving intravenous BPs since the age of three months, was treated under this protocol. The patient’s extraoral examination revealed blue sclerae, a concave profile, and upper jaw hypotrophy, while intraoral examination indicated mixed dentition, upper jaw contraction, and a right lateroposterior crossbite. Cephalometric analysis confirmed a Class III skeletal malocclusion, maxillary hypotrophy, and a Wits index of -3. Radiographic analysis showed enlarged pulp chambers in teeth affected by DI and agenesis of teeth 1.5, 3.5, and 4.5 (Figure 3A–E).

**Figure 4:**
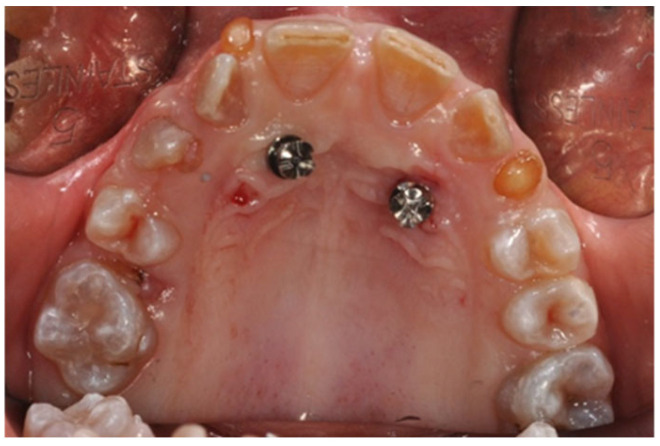
Miniscrew placement in OI patient.

**Figure 5:**
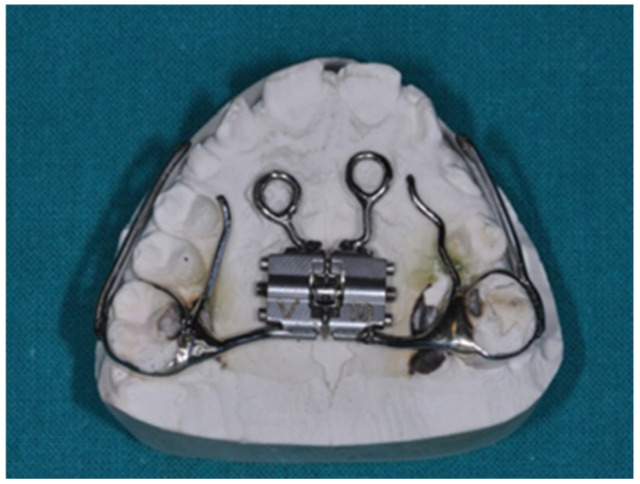
Customized hybrid MSE for hypihydrotic ED patient.

To address the anchorage challenge posed by DI, a Modified Sutural Expansion (MSE) approach was chosen, supplemented with palatal orthodontic miniscrews (Figure 4 and Figure 5). A precision silicone impression was taken, and cone beam computed tomography (CBCT) scans were performed to assess the cortical bone thickness before proceeding with miniscrew insertion. Despite the patient’s low bone thickness, 8 mm miniscrews (Sweden Signature, 1.8 mm diameter) were successfully placed to anchor the orthodontic device. Following antibiotic prophylaxis and BP suspension, the miniscrews were inserted under local anesthesia with a vasoconstrictor, and the patient’s parents were instructed on the activation protocol.

**Figure 6:**
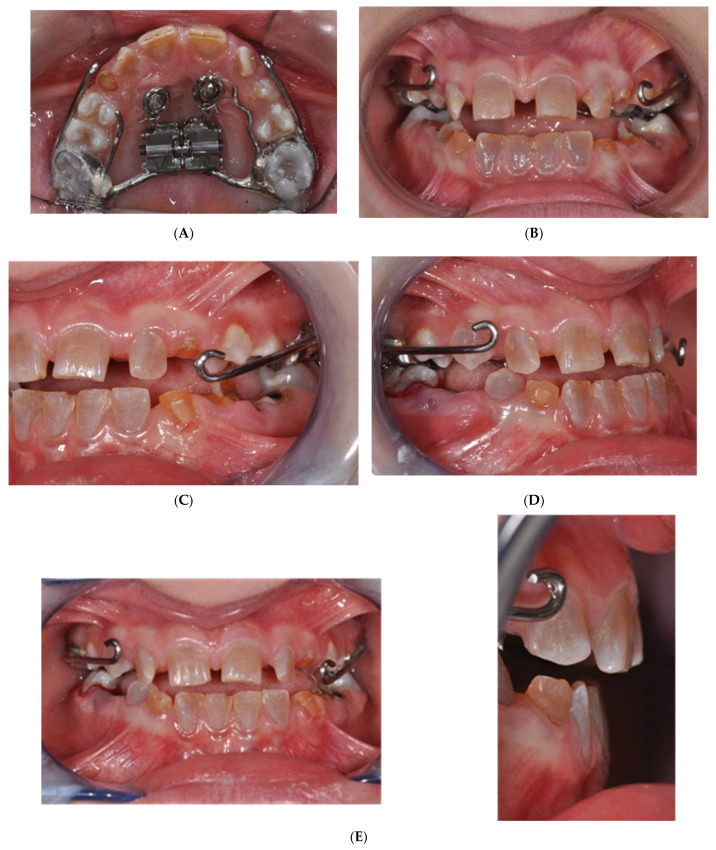
(**A**) MSE application for OI patient. (**B**) MSE application for OI patient. (**C**) MSE application for OI patient. (**D**) MSE application for OI patient. (**E**) MSE application for OI patient.

**Figure 7:**
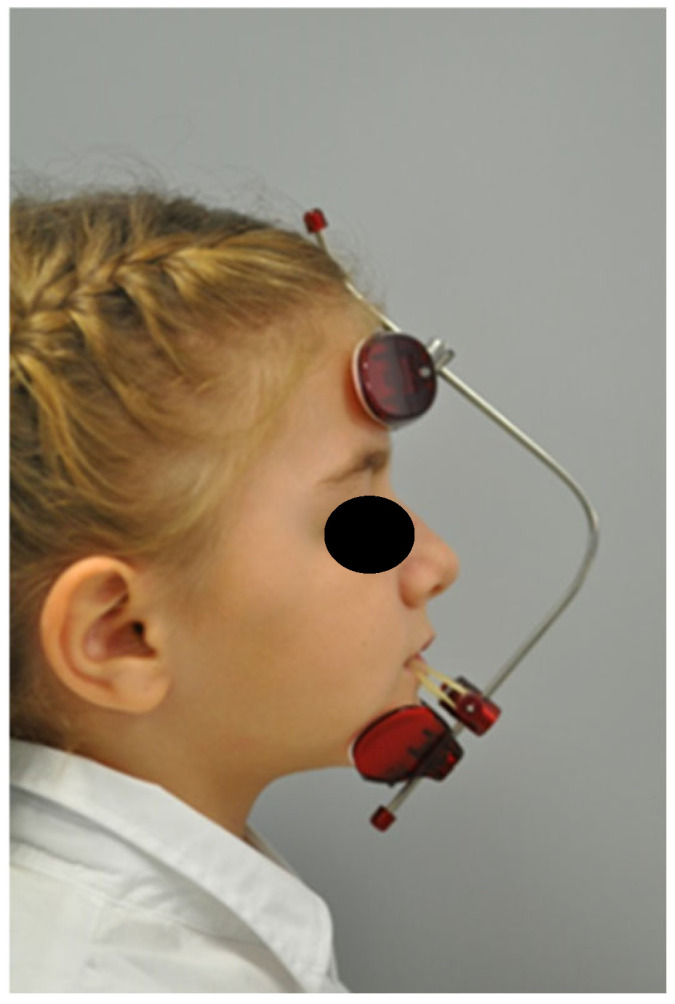
MSE and Petit’ Mask application for OI patient.

The MSE was activated twice daily for 15 days (Figure 6A–E), followed by the application of a Petit’s mask with 14 oz ¾ rubber bands for at least 14 h daily (Figure 7). The treatment showed significant improvement, with increased overjet (OJ) and overbite (OB) at three months. By nine months, the Class III malocclusion was completely corrected, with maxillary advancement, resolution of the crossbite, and completion of dental exchange (Figure 4).

At the 12-month follow-up, radiographic analysis showed delayed exfoliation of tooth 6.3, prompting the decision to extract this tooth to allow proper eruption of tooth 2.3. Cephalometric analysis confirmed significant improvement, with an increase in ANS and an ANB angle of 0. The patient’s orthodontic treatment continued with a Frankel Type III myofunctional appliance, although compliance remained a challenge due to the patient’s low cooperation.

Beyond dental interventions, the management of OI itself was also addressed through the introduction of a new experimental therapy with Denosumab, a monoclonal antibody targeting the RANK ligand to inhibit osteoclast formation and bone resorption. Unlike BPs, Denosumab has no long-term systemic accumulation and fewer side effects, making it a safer option for patients in the growing phase who are undergoing orthodontic treatment. Multidisciplinary collaboration was essential in this case, with the involvement of pediatricians, orthodontists, and oral surgeons to provide comprehensive care, monitor bone mineral density, and ensure the patient’s growth and development were not compromised during orthodontic treatment.

The multidisciplinary approach proved crucial in achieving the desired outcomes, addressing the patient’s dental and skeletal issues effectively while minimizing risks related to ongoing BP therapy. Follow-up visits continue to monitor the patient’s growth and provide guidance for future orthodontic interventions, ensuring optimal long-term outcomes for the patient.

The BP therapy was temporarily suspended to enable orthodontic treatment. This strategy was crucial for avoiding the inhibition of osteoclasts, which would have interfered with the tooth movement necessary for successful orthodontic correction.

The collaborative role of the pediatrician, orthodontist, and oral surgeon in managing the patient’s OI and DI conditions, coordinating the suspension of BP therapy, and monitoring bone density ensured the safe progression of orthodontic treatment. This comprehensive, team-based care approach was pivotal in managing the challenges associated with BP use and ensuring the patient’s safety during orthodontic treatment. Key details, such as treatment protocols, radiographic analysis, and follow-up timelines, were streamlined for easier understanding.

Each case was treated individually, and the orthodontic therapy was thoroughly discussed and planned in collaboration with the pediatrician to ensure optimal outcomes. As highlighted by Hartono et al. [12], our treatment successfully resolved malocclusion, with the clear opening of the palatine suture and the effective movement of the dental elements. This resulted in the resolution of crossbite issues without any significant side effects. The miniscrews remained stable throughout the treatment, showing no signs of mobility or subsidence. At the conclusion of therapy, the miniscrews were easily removed, along with the Modified Sutural Expansion (MSE) device.

Cone-beam computed tomography (CBCT) played a crucial role in guiding treatment for these patients, particularly in assessing bone structure before and during the procedure. CBCT imaging allowed for the precise placement of miniscrews, minimizing risks to permanent or unerupted teeth. It also helped identify areas of bone, primarily Type D1-D2 bone, which are typically more favorable for screw placement, ensuring that the treatment was both safe and effective.

Patient collaboration was essential for the success of the therapy. Active participation during treatment phases and diligent oral hygiene were vital in preventing complications such as miniscrew failure or interruption of orthodontic progress. To enhance patient compliance, the surgical procedure for miniscrew insertion followed the “Tell, Show, Do” approach. This method was particularly effective in increasing patient comfort and cooperation, especially among younger patients. The surgical technique was simple, effective, and well-received by all patients, contributing to the overall success of the treatment.

The orthodontic goals were achieved with significant improvements in dental arch dimensions and skeletal alignment. In particular, there was a measurable increase in overjet and overbite, and maxillary advancement was observed in cases of Class III malocclusion. Cephalometric analysis showed a marked improvement in ANS and ANB angles, with an average increase of 5 degrees in the skeletal relationship, suggesting effective maxillary growth. In terms of transverse expansion, the correction of the crossbite was confirmed through both clinical observation and radiographic analysis, with a wider palatal arch and resolution of the lateral crossbite at the 12-month follow-up.

The results remained stable over time, with no relapse of the corrected malocclusion during the follow-up period. Patients showed sustained improvements in dental alignment and skeletal structure. At the 1-year follow-up, the orthodontic results were stable in both OI and ED patients, with no indication of tooth migration or bite reversion. Orthopantomograms and lateral cephalograms confirmed that the maxillary expansion was maintained, and no negative effects were observed in permanent teeth eruption patterns.

**Figure 8:**
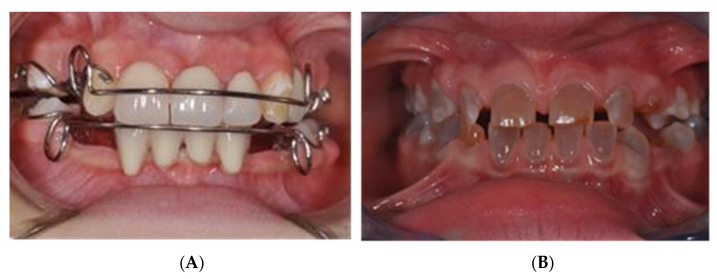
(**A**) Results in ED patient. (**B**) Results in OI patient.

To ensure long-term stability and optimal outcomes, we continue to provide regular follow-up appointments (Figure 8A,B). These visits allow for the monitoring of growth changes, any signs of relapse, and continued guidance on oral hygiene and orthodontic retention. In cases with reduced compliance, additional monitoring is scheduled to maintain the results achieved.

## 3. Discussion

Orthodontic therapy, particularly in patients with complex conditions like Osteogenesis Imperfecta (OI) and Ectodermal Dysplasia (ED), requires the careful management of anchorage and force application. The success of the treatment heavily depends on the precise use of Temporary Anchorage Devices (TADs), such as miniscrews, which are critical for managing malocclusions in these challenging cases [20,21,22,23]. However, treatment success is not without its limitations. One significant challenge is patient compliance, especially in pediatric cases. Successful orthodontic outcomes, including skeletal and dental movement, depend on the patient’s ability to maintain proper oral hygiene and follow the prescribed treatment plan. This is particularly important for ensuring the stability of miniscrews and achieving the desired orthodontic effects. In some cases, such as those involving ectodermal dysplasia or OI, the complexity of the disease further complicates management. For example, Dentinogenesis Imperfecta (DI), a common manifestation of OI, can interfere with normal tooth eruption and movement, necessitating a highly personalized orthodontic plan and multidisciplinary collaboration.

Another critical limitation lies in the quality and quantity of bone, especially in patients undergoing bisphosphonate therapy. As previously discussed, bisphosphonates reduce bone turnover, which can affect the integration and stability of TADs [24,25,26]. Despite this, the Maxillary Skeletal Expansion (MSE) procedure using miniscrews demonstrated positive results in these patients, albeit with careful monitoring and a temporary suspension of bisphosphonates during orthodontic treatment. Nevertheless, bone turnover rates, particularly in patients with endocrinological conditions or those undergoing systemic treatments, must be thoroughly evaluated before TAD insertion.

The results of this study were consistent with previous research on the use of TADs in orthodontics, particularly in patients with complex medical conditions. For instance, Ierardo et al. demonstrated the efficacy of MSE in growing patients with OI who were receiving bisphosphonate therapy, reporting no complications after a 1-year follow-up [27]. Similarly, other studies reported the success of skeletal anchorage in cases of maxillary transverse deficiency, highlighting its potential in cases where traditional dental anchorage would be insufficient or problematic. TADs are widely recognized as an important advancement in orthodontic treatment due to their ability to provide precise force application and stable anchorage, even in the absence of healthy or sufficient dental anchorage sites [28,29,30,31,32,33,34].

Additionally, the use of CBCT for the precise planning and placement of miniscrews in difficult anatomical areas—especially for patients with conditions like OI and ED—is strongly supported in the literature [35,36,37]. This imaging tool enables the careful assessment of bone thickness, interradicular spaces, and bone quality to ensure the most appropriate placement of TADs and avoid damage to adjacent structures or developing teeth [38,39]. However, challenges in managing rare diseases, such as dental anomalies and oral manifestations, continue to pose unique obstacles. These conditions often require specialized orthodontic care and careful monitoring of the patient’s medical history and growth patterns to achieve optimal outcomes.

For general pediatric dentists, the findings of this study have several important implications. As the use of TADs becomes more prevalent in orthodontics, it is essential that pediatric dentists be familiar with the indications, risks, and benefits of these devices, particularly when treating children with complex medical conditions. The ability to recognize when skeletal anchorage is necessary—either due to the absence of adequate dental anchorage or the presence of medical conditions that make traditional methods difficult—is critical. TADs provide an effective solution for managing complex malocclusions, maxillary transverse deficiency, and other skeletal issues, but their use must be carefully planned.

Additionally, for pediatric dentists who may encounter rare diseases like OI or ED, it is crucial to collaborate closely with specialists—including orthodontists, oral surgeons, and other healthcare providers such as neurologists or otolaryngologists—who can provide valuable insights into the patient’s overall health and treatment options. The management of Dentinogenesis Imperfecta (DI), a common dental manifestation in OI patients, requires specialized dental care to minimize damage to developing teeth while providing effective orthodontic treatment.

Informed decision-making for the use of TADs involves a multidisciplinary approach, including the pediatrician, orthodontist, and any relevant specialists. CBCT imaging is an invaluable tool for precise diagnosis and treatment planning, especially in cases with complex dental or bone abnormalities.

Ultimately, understanding the unique challenges posed by rare diseases in pediatric orthodontics will enable general practitioners to offer tailored care that addresses both dental and medical needs. By staying informed about emerging technologies like TADs and integrating them into personalized treatment plans, pediatric dentists can significantly enhance treatment outcomes and the overall quality of life for patients with these complex conditions.

### Limitation of the Study

While this study provided important insights, future research with larger sample sizes is necessary to further evaluate the efficacy of orthodontic therapy supported by MSE across a broader range of cases. This should include investigating its impact on special needs patients and those facing challenges related to anchorage in various clinical situations in pediatric dentistry. These factors should be addressed in future studies to refine treatment protocols and enhance patient outcomes.

## 4. Conclusions

This study aimed to highlight the significant clinical advantages of orthodontic miniscrews, particularly in challenging pediatric cases involving rare systemic or dental conditions. Our results emphasized the effectiveness of hybrid TAD-assisted expanders, which offer specific benefits in maxillary expansion for patients with complex conditions such as Osteogenesis Imperfecta (OI) and Ectodermal Dysplasia (ED). These devices provide a safe, efficient, and minimally invasive alternative to traditional orthodontic methods, ensuring better stability and precision in treatment outcomes, even when standard dental anchorage options are unavailable.

The successful application of these devices requires more than just technical proficiency. Multidisciplinary collaboration is essential to ensure optimal care. A comprehensive treatment plan should involve a network of specialists, including the orthodontist, pediatrician, oral surgeon, dental hygienist, and the patient’s family. This collaborative approach ensures that the patient’s medical, dental, and psychosocial needs are addressed throughout the treatment process, leading to more effective and personalized outcomes. The integration of TADs into orthodontic care represents a significant advancement in the treatment of rare and complex pediatric conditions, demonstrating their role in improving treatment success rates and minimizing risks.

## Data Availability

Data available upon request due to restrictions. The data presented in this study are available upon request. The data are not publicly available for privacy reasons.
